# Non-alcoholic fatty liver disease and coexisting depression, anxiety and/or stress in adults: a systematic review and meta-analysis

**DOI:** 10.3389/fendo.2024.1357664

**Published:** 2024-04-16

**Authors:** Sue Shea, Christos Lionis, Chris Kite, Lukasz Lagojda, Olalekan A. Uthman, Alexander Dallaway, Lou Atkinson, Surinderjeet S. Chaggar, Harpal S. Randeva, Ioannis Kyrou

**Affiliations:** ^1^ Warwick Medical School, University of Warwick, Coventry, United Kingdom; ^2^ Warwickshire Institute for the Study of Diabetes, Endocrinology and Metabolism (WISDEM), University Hospitals Coventry and Warwickshire NHS Trust, Coventry, United Kingdom; ^3^ Laboratory of “Health and Science” School of Medicine, University of Crete, Heraklion, Greece; ^4^ Department of Health, Medicine and Caring Sciences, University of Linkoping, Linkoping, Sweden; ^5^ Department of Nursing, Frederick University, Nicosia, Cyprus; ^6^ School of Health and Society, Faculty of Education, Health and Wellbeing, University of Wolverhampton, Wolverhampton, United Kingdom; ^7^ Chester Medical School, University of Chester, Shrewsbury, United Kingdom; ^8^ Centre for Sport, Exercise and Life Sciences, Research Institute for Health & Wellbeing, Coventry University, Coventry, United Kingdom; ^9^ Clinical Evidence-Based Information Service (CEBIS), University Hospitals Coventry and Warwickshire NHS Trust, Coventry, United Kingdom; ^10^ Division of Health Sciences, Warwick Centre for Global Health, Warwick Medical School, University of Warwick, Coventry, United Kingdom; ^11^ iPrescribe Exercise Digital Ltd (EXI), London, United Kingdom; ^12^ Sowe Valley Primary Care Network, Forum Health Centre, Coventry, United Kingdom; ^13^ Institute of Cardiometabolic Medicine, University Hospitals Coventry and Warwickshire NHS Trust, Coventry, United Kingdom; ^14^ Aston Medical School, College of Health and Life Sciences, Aston University, Birmingham, United Kingdom; ^15^ College of Health, Psychology and Social Care, University of Derby, Derby, United Kingdom; ^16^ Laboratory of Dietetics and Quality of Life, Department of Food Science and Human Nutrition, School of Food and Nutritional Sciences, Agricultural University of Athens, Athens, Greece

**Keywords:** non-alcoholic fatty liver disease, NAFLD, NASH, mental health, depression, anxiety, stress

## Abstract

**Background:**

Non-alcoholic fatty liver disease (NAFLD) is a common chronic liver disease, affecting 25-30% of the general population globally. The condition is even more prevalent in individuals with obesity and is frequently linked to the metabolic syndrome. Given the known associations between the metabolic syndrome and common mental health issues, it is likely that such a relationship also exists between NAFLD and mental health problems. However, studies in this field remain limited. Accordingly, the aim of this systematic review and meta-analysis was to explore the prevalence of one or more common mental health conditions (i.e., depression, anxiety, and/or stress) in adults with NAFLD.

**Methods:**

PubMed, EBSCOhost, ProQuest, Ovid, Web of Science, and Scopus were searched in order to identify studies reporting the prevalence of depression, anxiety, and/or stress among adults with NAFLD. A random-effects model was utilized to calculate the pooled prevalence and confidence intervals for depression, anxiety and stress.

**Results:**

In total, 31 studies were eligible for inclusion, involving 2,126,593 adults with NAFLD. Meta-analyses yielded a pooled prevalence of 26.3% (95% CI: 19.2 to 34) for depression, 37.2% (95% CI: 21.6 to 54.3%) for anxiety, and 51.4% (95% CI: 5.5 to 95.8%) for stress among adults with NAFLD.

**Conclusion:**

The present findings suggest a high prevalence of mental health morbidity among adults with NAFLD. Given the related public health impact, this finding should prompt further research to investigate such associations and elucidate potential associations between NAFLD and mental health morbidity, exploring potential shared underlying pathophysiologic mechanisms.

**Systematic review registration:**

https://www.crd.york.ac.uk/prospero/, identifier CRD42021288934.

## Introduction

Non-alcoholic fatty liver disease (NAFLD) develops as a result of excess accumulation of fat in hepatocytes, which is unrelated to excess alcohol intake, and extends from simple steatosis to non-alcoholic steatohepatitis (NASH) with or without fibrosis that may lead to liver failure and even hepatocellular carcinoma (1–3). NAFLD currently constitutes the most prevalent chronic liver disease worldwide with prevalence rates of up to 25-30% among the general adult population (1–3). Furthermore, NAFLD is frequently linked to the metabolic syndrome which represents a cluster of interrelated cardio-metabolic conditions associated with central obesity, and obesity-related insulin resistance [i.e., type 2 diabetes mellitus (T2DM), hypertension and dyslipidemia] (4). Indeed, it is reported that approximately 85% of individuals with NAFLD exhibit at least one element of the metabolic syndrome (5), with the prevalence of NAFLD among individuals with obesity reaching 70-90% (2, 3, 6). In addition, it is reported that future generations are at risk of a ‘second wave’ of metabolic liver disease, in the form of NAFLD, owing to potential early-onset as an impact of weight issues during childhood (7).

Owing to these associations with obesity and the metabolic syndrome, NAFLD is often referred to as the hepatic manifestation of metabolic syndrome (8–11). Of note, to highlight these links and to more accurately describe the pathophysiology of NAFLD, renaming this condition to metabolic dysfunction-associated fatty liver disease (MAFLD) or metabolic dysfunction-associated steatotic liver disease (MASLD) has been recently proposed (12, 13). Indeed, as reported by the European Association for the Study of the Liver (14), the term ‘MASLD’ is reflective of patients with hepatic steatosis who experience more than one of five cardiometabolic risk factors, and thus is considered to be less stigmatizing and a preferred nomenclature as opposed to the term ‘NAFLD’. Taking into account these newer proposed terms for NAFLD, it is noteworthy that, in addition to introducing the new nomenclatures of MAFLD and MASLD in the scientific literature, the definitions of these nosologies are also redefined based on specific diagnostic criteria for each term (15–18). As such, whereas the diagnosis of NAFLD requires the exclusion of alternative etiologies of steatosis/steatohepatitis (e.g., alcoholic or viral hepatitis), the diagnosis of both MAFLD and MASLD acknowledges that in such patients a combination of dysmetabolic and other (e.g. alcohol-related) pathophysiologic components may contribute to the underlying hepatic nosology (15–18). Accordingly, these conditions are diagnosed based primarily on the presence of metabolic dysfunction rather than on the exclusion of other causes of steatosis/steatohepatitis. Thus, MAFLD is defined as steatosis which is detected - either by imaging or blood biomarkers/scores or histology - in the presence of at least either obesity, and/or T2DM, or at least two out of seven predefined dysmetabolic risk abnormalities (relating to waist circumference, blood pressure, plasma triglycerides, plasma HDL-cholesterol, plasma high-sensitivity C-reactive protein, prediabetes, and the homeostatic model assessment for insulin resistance score) in those adults who are lean (normal weight by ethnic-specific BMI criteria) and do not have T2DM (15, 16, 18). Similarly, MASLD is defined as the presence of steatotic liver disease combined with at least one of five predefined cardio-metabolic criteria relating to BMI, fasting plasma glucose levels, blood pressure, plasma triglycerides, and plasma HDL-cholesterol (17). From these definitions, it is evident that, despite the significant overlap (>95% of adult patients previously diagnosed as having NAFLD also fulfil the MASLD diagnostic criteria) (19), the terms NAFLD, MAFLD and MASLD cannot always be applied interchangeably, whilst there are also concerns regarding whether the clinical evidence accumulated for NAFLD can be directly extrapolated to MAFLD and MASLD (20). Indeed, following the introduction of the term MASLD, researchers have called for more flexible editorial conduct regarding the proposed MASLD nomenclature, since these three nosologies/terms are defined differently and, thus, accurate distinction between NAFLD, MAFLD, and MASLD is important for the accuracy of the relevant scientific literature (20). To address issues relating to the different definitions of NAFLD, MAFLD and MASLD, in the present systematic review the NAFLD terminology has been retained since the accumulated evidence of interest has been primarily accumulated under the NAFLD nomenclature/definition.

NAFLD often remains asymptomatic for a lengthy duration, hence representing a ‘silent epidemic’ (21). However, NAFLD constitutes a significant risk factor for cardiovascular disease (CVD), which is reported as the most common cause of mortality in this patient population (21, 22). In parallel to the data highlighting NAFLD as an evolving epidemic, growing evidence also suggests direct associations between common mental health issues, such as depression, anxiety and chronic stress, and the metabolic syndrome (23, 24). Based on the strong overlap between NAFLD and the metabolic syndrome, it seems likely that such associations may also be observed in individuals with NAFLD, potentially with shared underlying mechanisms that create a feed-forward vicious cycle between NAFLD and such mental health morbidity (12). However, further research is required to fully clarify the complete spectrum of such potential associations. Furthermore, it is plausible that certain features associated with NAFLD, such as lack of awareness regarding the condition, fatigue, and perceived stigma (13, 25–27), may result in feelings of isolation and loneliness (28), which, in turn, may have a further impact on mental health and have been reported to be associated with cardio-metabolic disorders linked to NAFLD, including obesity, T2DM, metabolic syndrome, and CVD (29, 30).

In this context, research addressing potential mental health issues in individuals with NAFLD warrants attention. However, despite previous systematic reviews which have investigated links between psychological health and NAFLD and associations with depression (31–33), such issues remain relatively under-recognized in clinical practice. Indeed, a systematic review by Macavie et al. (32) draws attention to depression and anxiety as the most relevant emotional factors among individuals with NAFLD/NASH, suggesting that such conditions may be regarded as cognitive-behavioral in nature with lifestyle modification representing the most effective management (32). Furthermore, additional systematic reviews - albeit with a low number (up to ten) of included eligible studies (31, 33) - have demonstrated an association between NAFLD and depression.

Given the limited but growing data in this field, the present systematic review and meta-analysis aimed to explore the prevalence of one or more common mental health conditions of interest (i.e., depression, and/or anxiety, and/or stress) in adults with NAFLD, and to identify relevant gaps and weaknesses within the existing literature.

## Methodology

### Search strategy and study selection

This systematic review was prepared in accordance with the Preferred Reporting Items for Systematic Reviews and Meta-Analyses (PRISMA) Guidelines (34), and was registered with the International Prospective Register of Systematic Reviews (PROSPERO Reference Number: CRD42021288934).

Inclusion criteria were any study (observational or interventional) published as a scientific paper reporting the prevalence of at least one of the three mental health conditions of interest (i.e., depression, anxiety, or chronic psychological stress) in adults (male and female) aged over 18 years with a diagnosis of NAFLD.

A search was conducted in relation to NAFLD and mental health utilizing the PubMed, EBSCOhost, ProQuest, Ovid, Web of Science, and Scopus databases. The search terms applied for the PubMed database included the following: ((metabolic associated fatty liver disease[Title/Abstract] OR MAFLD OR metabolic dysfunction associated fatty liver disease[Title/Abstract] OR NAFLD[MeSH Terms] OR NAFLD OR non-alcoholic fatty liver disease[Title/Abstract] OR non-alcoholic steatohepatitis[Title/Abstract] OR NASH)) AND ((mental health[MeSH Terms] OR mental health[Title/Abstract] OR “mental health” OR “mental well-being” OR “mental wellbeing” OR depression[MeSH Terms] OR depression[Title/Abstract] OR major depressive disorder[MeSH Terms] or major depressive disorder[Title/Abstract] OR major depression[Title/Abstract] OR MDD OR anxiety[MeSH Terms] OR anxiety[Title/Abstract] OR generalized anxiety disorder[MeSH Terms] OR generalized anxiety disorder[Title/Abstract] OR generalized anxiety disorder[Title/Abstract] OR stress, psychologic[MeSH Terms] OR disorder, mood[MeSH Terms] OR distress[Title/Abstract])). This search string was applied and adapted to the syntax of all of the utilized databases (**Supplementary Table 1**).

The searches were conducted by LL and the results of the searches were imported into Covidence systematic review software V2.0 (Veritas Health Innovation, Melbourne, Australia). Following removal of duplicates, title and abstract screening was completed by SS, LL and CK. No publication date restriction was adopted for the timeframe of the search strategy (no publication date restriction up to 2022). Full-text screening was performed by SS and LL, with any disputes being resolved by the inclusion of a third reviewer (CK).

### Data extraction and quality assessment

Data (including country, year, study design, number of participants, mental health measures, NAFLD diagnosis, gender, and age) were independently extracted by two reviewers (SS, LL), with the outcome of interest being the prevalence of depression, anxiety, and/or stress. Any disagreements or possible input errors were checked and resolved via discussion between the two reviewers.

Risk of bias assessment was performed by SS and LL using the Covidence systematic review software V2.0 which utilizes a standard template based on the Cochrane Risk of Bias version 1 tool. The assessment criteria were amended within Covidence to reflect risk of bias assessment for non-randomized studies (RoBaNS) (35). Any disputes were settled by a third reviewer (CK). The categories assessed were selection of participants, confounding variables, exposure measurements, selective outcome reporting, incomplete outcome data, and other sources of bias. Author judgement for risk of bias was rated as high, low, or unclear for each category (**Supplementary Figure 1**).

### Statistical analysis

The Freeman-Tukey variant of the arcsine square root transformation was applied in order to normalize the raw prevalence estimates obtained from each included study; an approach commonly used for the pooling of proportions (36). For the performed meta-analyses, the DerSimonian-Laird random-effects model was utilized; a methodology frequently adopted in anticipation of discrepancies in population demographics, research techniques, and study environments (37). The heterogeneity amongst studies was evaluated by examining the forest plots, and by applying the chi-squared test for heterogeneity, setting a statistical significance level of P ≤ 0.10, as well as the use of the *I* ^2^ statistic, with a 50% value indicative of moderate heterogeneity (38), and a 75-100% value representing considerable heterogeneity (39).

Subsequent to the primary analyses, additional subgroup analyses were also conducted, differentiated by the types of validated instruments used to deduce prevalence estimates. The potential for reporting bias was examined using a funnel plot, a graphical tool typically used to assess the presence of publication bias in systematic reviews (40). The robustness of the meta-analysis results were evaluated using a leave-one-study-out sensitivity analysis (41). In addition, to assess the influence of individual studies on the overall meta-analysis results and their contribution to heterogeneity, we utilized Baujat plots. This graphical tool plots the contribution of each study to the overall heterogeneity against its influence on the overall result (42).

## Results

**Figure 1:**
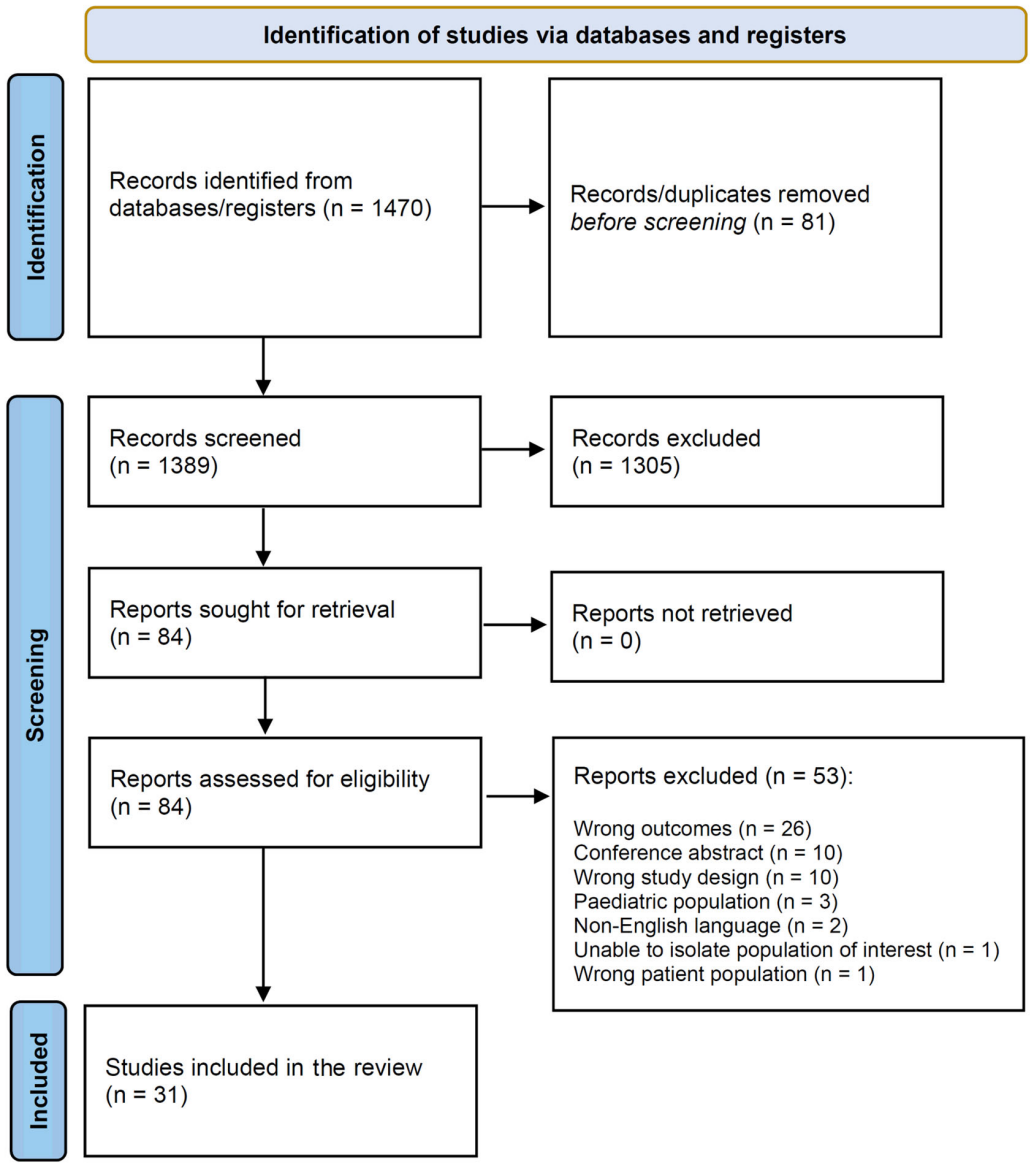
PRISMA flow chart for the present systematic review.

A total of 1470 studies were identified from the performed database searches and were then imported to Covidence where 81 duplicates were removed, thus resulting in 1389 studies for title and abstract screening. Following title and abstract screening, 1305 studies were considered irrelevant, leading to an initial total of 84 studies going forward for full text review. During full text review, 53 studies were excluded with reasons (**Figure 1**), resulting in a total of 31 studies eligible for inclusion.

For the 31 studies included in this systematic review, NAFLD was defined by various means including liver biopsy, ultrasonography/evidence of ultrasound, hepatic steatosis index, pathology and/or radiologic testing, computed tomography, magnetic resonance imaging, and self-reported physician diagnosis (**Table 1**). From the 31 included studies, 18 studies (58%) measured only depression (44, 49–53, 57, 58, 61–70), one study measured only anxiety (55), 10 studies (32%) measured depression and anxiety (43, 45–48, 56, 60, 71–73), one study measured only stress (54), and one study measured stress and anxiety (59). In these studies, the mental health conditions of interest were identified by validated measures (including DSM-IV and ICD-10) in 17 studies (44, 46, 48, 49, 53, 54, 56–59, 63, 65–68, 70, 73), self-reported in six studies (43, 47, 55, 61, 62, 69), or identified by other diagnosis (e.g., medical history) in eight studies (45, 50–52, 60, 64, 71, 72). Characteristics of the included studies are presented in **Table 1**.

**Table 1:** Study characteristics of the 31 eligible studies on non-alcoholic fatty liver disease (NAFLD) and coexisting depression, anxiety and/or stress in adults which are included in the present systematic review.

Study (year)	Country	Study Design	Participant Characteristics	Mental Health Assessment Method	NAFLD Diagnosis	Summarized Main Study Findings
Balp et al. (2019) (43)	European	Cross-Sectional	Total sample: n = 184 (male: 57.1%) Age: 54.5 (13.1) years Depression: n = 57 Anxiety: n = 59	Self-Reported	Self-reported physician diagnosis	Depression and anxiety diagnosis was greater in the NASH cohort, compared to the matched general population, with a significant burden to HRQoL.
Canivet et al. (2020) (44)	France	Prospective cohort study	Total sample: n =388* (female: 81%) Age: 40 (30-50) *A sub-sample of 183 patients were selected from the initial sample of 388 and were tested for depression. Depression: n = 62 (BDI) 83 (HADS)	BDI HADS	Liver Biopsy	Participants with severe obesity had more severe BED and depression compared to lean individuals, independent of NAFLD severity.
Castellanos-Fernández et al. (2021) (45)	Cuba	Cross-Sectional	Total sample: n = 221 (female: 67.9%) Age: 54 (11.3) years Depression: n = 86 Anxiety: n = 124	Other diagnosis (e.g. medical history)	Liver biopsy or imaging	Fatigue, anxiety, depression and abdominal pain represented the strongest independent predictors of HRQoL among participants.
Choi et al. (2021) (46)	South Korea	Retrospective Cross-Sectional	Total sample: n = 7,846 (male: 78.63%) Age: 50.5 (10) years Depression: n = 335 State Anxiety: n = 541 Trait Anxiety: n = 162	BDI STAI	Ultrasonography	NAFLD was significantly and independently associated with depression. Steatosis stage had significant associations with both state anxiety and trait anxiety in women.
Doward et al. (2021) (47)	USA	Qualitative	Total sample: n = 43 (female: 66.65%) Age: 53.25 (10.2) years Depression: n = 13 Anxiety: n = 8	Self-Reported	Liver biopsy or phenotypic diagnosis	Depression was one of the most frequently reported comorbidities (>25% mentioned feeling depressed and anxious due to NASH).
Elwing et al. (2006) (48)	USA	Case-control study	Total sample: n = 36 (female: 58.3% Age: 48.8 (2.01) years Depression: n = 20 Anxiety: n = 18	DSM-IV	Liver Biopsy	Lifetime rates of major depressive disorder and general anxiety disorder were significantly increased in patients with NASH, and were associated with advanced histological hepatic abnormalities.
Fillipovic, Markovic & Duric (2018) (49)	Serbia	Case-control study	Total sample: n = 40 (male: 55%) Age: 47.88 (6.07) years Depression: n = 33	HAM-D	Abdominal ultrasound	Patients with NAFLD had a higher risk of depression compared to those without.
Forlano et al. (2021) (50)	UK	Service Evaluation Project	Total sample: n = 81 (female: 61.73%) Age (with BEDs): 52 (45-57.5) years Age (without BEDs): 59 (49-63) years Depression: n = 15	Other Diagnosis e.g. Medical History	Not reported	Participants with BED experienced more frequent depression than those without.
Glass et al. (2021) (51)	USA	Intervention Study	Total sample: n = 248 (female: 54%) Age: 53.5 (44-62) years Depression: n = 100	Other Diagnosis e.g. Medical History	Ultrasound, computed tomography, or magnetic resonance imaging	Depression was independently associated with high-risk behaviors (e.g. unhealthy diet and sedentary behavior) among people with NAFLD.
Huang et al. (2021) (52)	China	Cross-Sectional	Total sample: n = 5,181 (male: 65.8%) Age: 43.8 (13.3) years Depression: n = 135	Other Diagnosis e.g. Medical History	Ultrasound, computed tomography, and magnetic resonance imaging in 24 months or liver biopsies in 36 months.	Depression, and factors such as disease severity, CVD and diabetes, influenced HRQoL based on the CLDQ-NAFLD.
Jung et al. (2019) (53)	South Korea	Cross-sectional	Total sample: n = 31,635 (male: 77.38%) Age: 41.25 (7.15) years Depression: n = 2,870	CES-D	Abdominal ultrasound	NAFLD, both in terms of presence and severity was associated with depressive symptoms.
Kang et al. (2020) (54)	South Korea	Cross-Sectional	Total sample: n = 47,538 (male: 76.6%) Age: 42 (9.1) years Stress: n = 36,555	PSI	Ultrasound	Perceived stress levels were associated with the NAFLD prevalence, even after controlling for behavioral metabolic, & socioeconomic, factors (stronger association in men, and in participants with obesity).
Khoonsari et al. (2017) (55)	Iran	Cross-Sectional	Total sample: n = 206 (male: 52.9%) Age: 41.2 (8.3) years Anxiety: n = 181	Self-Reported	Ultrasonography	Anxiety and gastrointestinal problems were common in patients with NAFLD.
Labenz et al. (2020) (56)	Germany	Retrospective cohort study	Total sample: n = 19,871 (male: 57.5%) Age: 58.5 (14.2) years Depression: n = 4,173 Anxiety: n = 1,590	ICD-10	Not specified	NAFLD was identified as an independent risk factor for depression and anxiety.
Lee & Park (2021) (57)	Korea	Cross-Sectional	Total sample: n = 4,688 (female 61.6%) Age: 48.25 (0.75) years Depression: n = 422	PHQ-9	Hepatic steatosis index	Adults with depression had a higher risk of NAFLD, with depression also being associated to insulin resistance.
Lee et al. (2013) (58)	USA	Cross-Sectional	Total sample: n = 497 (female: 55%) Age: 49.62 (0.72) years Depression: n = 148	PHQ-9	NAFLD defined by the absence of any other causes of CLD	Depression was not found to be independently associated with NAFLD at a population level after controlling for other confounding factors.
Magalhaes et al. (2020) (59)	Brazil	Case-control study	Total sample: n = 26 (female: 89.1%) Age: 37 (8.9) years Anxiety: n = 21 Stress: n = 6	HAM-A LSSI	Ultrasonography	Findings did not identify significant associations between NAFLD and anxiety or stress, although all participants with NAFLD had some level of anxiety. No significant association between NAFLD and stress was identified.
Moon et al. (2021) (60)	USA	Prospective cohort study	Total sample: n = 3,474 (female: 58.9%) Age: 56.9 (12.96) years Depression: n = 1,333 Anxiety: n = 925	Other Diagnosis e.g. Medical History	Liver biopsy and/or pragmatic case definitions	Opioid use was identified in 1 out of 5 patients with NAFLD and was more common in those with depression, anxiety, and severe liver disease.
Patel et al. (2017) (61)	Australia	Prospective cohort study	Total sample: n = 95 (male: 61%) Age: 59.6 (9.4) years Depression: n = 42	Self-Reported	Ultrasound	Adults with NAFLD and T2DM had at least two other chronic conditions, with the most common being metabolic syndrome and self-reported depression.
Patel et al. (2017) (62)	Australia	Cross-Sectional	Total sample: n = 151 (male: 63.6%) Age: 60.7 (10.3) years Depression: n = 72	Self-Reported	Ultrasound	Self-reported depression was highly prevalent and more common in those with moderate alcohol consumption.
Sayiner et al. (2020) (63)	USA	Cross-sectional	Total sample: n = 1,980,950 (female: 54.7%) Age: 70 (11.11) years Depression: n = 188,307	ICD-10	ICD-9/ICD-10 Codes	Depression was among the most common extra-hepatic diseases identified.
Shaheen et al. (2021) (64)	United Kingdom	Retrospective cohort study	Total sample: n = 19,053 (female: 54.7%) Age: 54.1 (12.7) years Depression: n = 3,061	Other Diagnosis e.g. Medical History	Read Codes	No significant difference in liver disease progression among patients with NAFLD and ALD in relation to major depressive disorder.
Surdea-Blaga & Dumitraşcu (2011) (65)	Romania	Cross-Sectional	Total sample: n = 63 (female: 60.3%) Median Age: 46.4/50.1 years (men/women) Depression: n = 36	BDI	Abdominal Ultrasound	No significant relationship between depression/anxiety and NAFLD. Anxiety and depression are common in the studied region.
Takahashi et al. (2017) (66)	Japan	Retrospective cohort study	Total sample: n = 24 (female: 100%) Age: 54 (47-61) years Depression: n = 1	CES-D	Ultrasonography	Potential association between decreased brain activity and NAFLD, regardless of depression.
Tomeno et al. (2015) (67)	Japan	Retrospective cohort study	Total sample: n = 258 (male: 53.1%) Age: 48.6 (13.25) years Depression: n = 32	DSM-IV	Liver biopsy	The comorbid state of MDD was associated with more severe histological steatosis and worse treatment outcomes in NAFLD.
Tutunchi et al. (2021) (68)	Iran	Case-control study	Total sample: n = 95 (female: 56.8%) Age: 48.8 (5.9) years Depression: n = 44	BDI	Ultrasonography	Higher prevalence of depression in those with NAFLD, compared to those without NAFLD.
Weinstein et al. (2011) (69)	USA	Cross-Sectional	Total sample: n = 184 (female: 69.4%) Age: 46.7 (11.2) years Depression: n = 50	Self-Reported	Pathology and/or radiologic testing	Patients with NAFLD and HCV had higher depression prevalence compared to individuals with HBV and the depression rates among the general population.
Yang et al. (2021) (70)	USA	Cross-Sectional	Total sample: n = 595 (female: 53.2%) Age: 59.9 (0.7) years Depression: n = 65	PHQ-9	Liver steatosis in the absence of possible secondary causes of fatty liver.	Depression was an independent predictor for MAFLD risk, with a positive relationship between depression and MAFLD in middle‐aged and older adults.
Younossi et al. (2019) (71)	USA	Cross-Sectional	Total sample: n = 1,338 (female: 53.1%) Age: 57 (8.9) years Depression: n = 339 Anxiety: n = 260	Other Diagnosis e.g. Medical History	Histologic evidence	NASH was associated with significant impairment on patient reported outcomes and well-being.
Younossi et al. (2020) (72)	USA	Cross-Sectional	Total sample: n = 1,222 (female: 56.7%) Mean Age: 57.8 years Depression: n = 272 Anxiety: n = 335	Other Diagnosis e.g. Medical History	Liver biopsy	Depression or a nervous system disorder were associated with fatigue and increased likelihood to report pruritus.
Youssef et al. (2013) (73)	USA	Cross Sectional	Total sample: n = 567 (female: 67%) Age: 48 (1.1) years Depression: n = 80 Anxiety: n = 143	HADS	Histological diagnosis of NAFLD	Subclinical and clinical depression was noted in 53% and 14% of patients, respectively. Increased severe depression symptoms were associated with a greater likelihood of severe hepatocyte ballooning.

ALD, Alcoholic Liver Disease; BDI, Beck Depression Inventory; BED, binge eating disorder; BMI, Body Mass Index; CES-D, Centre for Epidemiological Studies-Depression; CLDQ, Chronic Liver Disease Questionnaire; CVD, Cardiovascular Disease; DSM-IV, Diagnostic and Statistical Manual of Mental Health Disorders; HADS, Hospital Anxiety and Depression Scale; HAM-A, Hamilton Anxiety Rating Scale; HAM-D, Hamilton Depression Rating Scale; HBV, Hepatitis B; HCV, Hepatitis C; HRQoL, Health-Related Quality of Life; ICD, International Classification of Diseases; LSSI, Lipp’s Stress Symptoms Inventory; MAFLD, Metabolic Dysfunction-Associated Fatty Liver Disease; NAFLD, non-alcoholic fatty liver disease; PHQ-9, Patient Health Questionnaire; PSI, perceived stress inventory; STAI, State-Trait Anxiety Inventory; T2DM, Type 2 Diabetes Mellitus. Age is reported as mean (Standard Deviation or range), or median (Interquartile Range) based on available data reported by each study.

### Assessment of risk of bias

**Figure 2:**
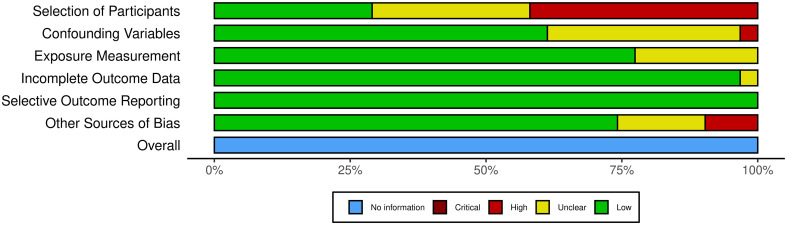
Risk of bias assessment of the included studies.

Judgements regarding risk of bias are presented in **Figure 2**, whilst further information is available in **Supplementary Figure 1**.

Selection bias was identified in 13 studies (42%). The main support for judgement was that for these studies, patients had been recruited from a single center and therefore findings may not be representative of the general patient population with NAFLD. Selection bias was judged to be low for nine studies (29%) and unclear for nine studies (29%). For confounding factors, 19 studies (61%) were judged to have a low risk of bias, since these had been controlled for within analyses. The remaining studies were judged as having an unclear risk for 11 studies (35.4%) and high risk for one study (3.2%). Risk of bias was judged as low for intervention (exposure) measurement for 24 (77.4%) studies, with the remaining seven studies (22.5%) judged as unclear owing to the use of self-report measures. Low risk of bias was also reported for incomplete outcome data in 30 (96.7%) studies, with one study identified as unclear. Selective outcome reporting was judged as being low risk of bias for all included studies. When other sources of bias were assessed, 22 studies were judged as low risk (70%), five studies (16.1%) were judged as unclear, whilst four (12.9%) studies were rated as having a high risk of bias.

### Depression

In total, 28 of the included studies measured depression, with the total number of participants amounting to 2,079,270. Validated instruments were used to measure depression in 15 studies (44, 46, 49, 53, 56–59, 63, 65–68, 70, 73), while self-report and other diagnosis (e.g., medical history) were used in five (43, 47, 61, 62, 69) and eight studies (45, 50–52, 60, 64, 71, 72), respectively. Of these studies, 11 were from the USA (47, 48, 51, 58, 60, 63, 69–73), resulting in a total of 1,989,154 participants from this geographical region. However, the majority of USA participants were recruited for one particular study involving 1,980,950 individuals (63).

It should be noted that one study (44) had utilized both the Beck Depression Inventory (BDI) and the Hospital Anxiety and Depression Scale (HADS) to measure depression, but we included only the data from the BDI within the primary analysis for pooled prevalence of depression, as this had gleaned a higher prevalence when the authors reported mild depression in addition to moderate to severe. When the data were analyzed by sub-groups, on the basis of individual validated measures, both the BDI and HADS were included.

**Figure 3:**
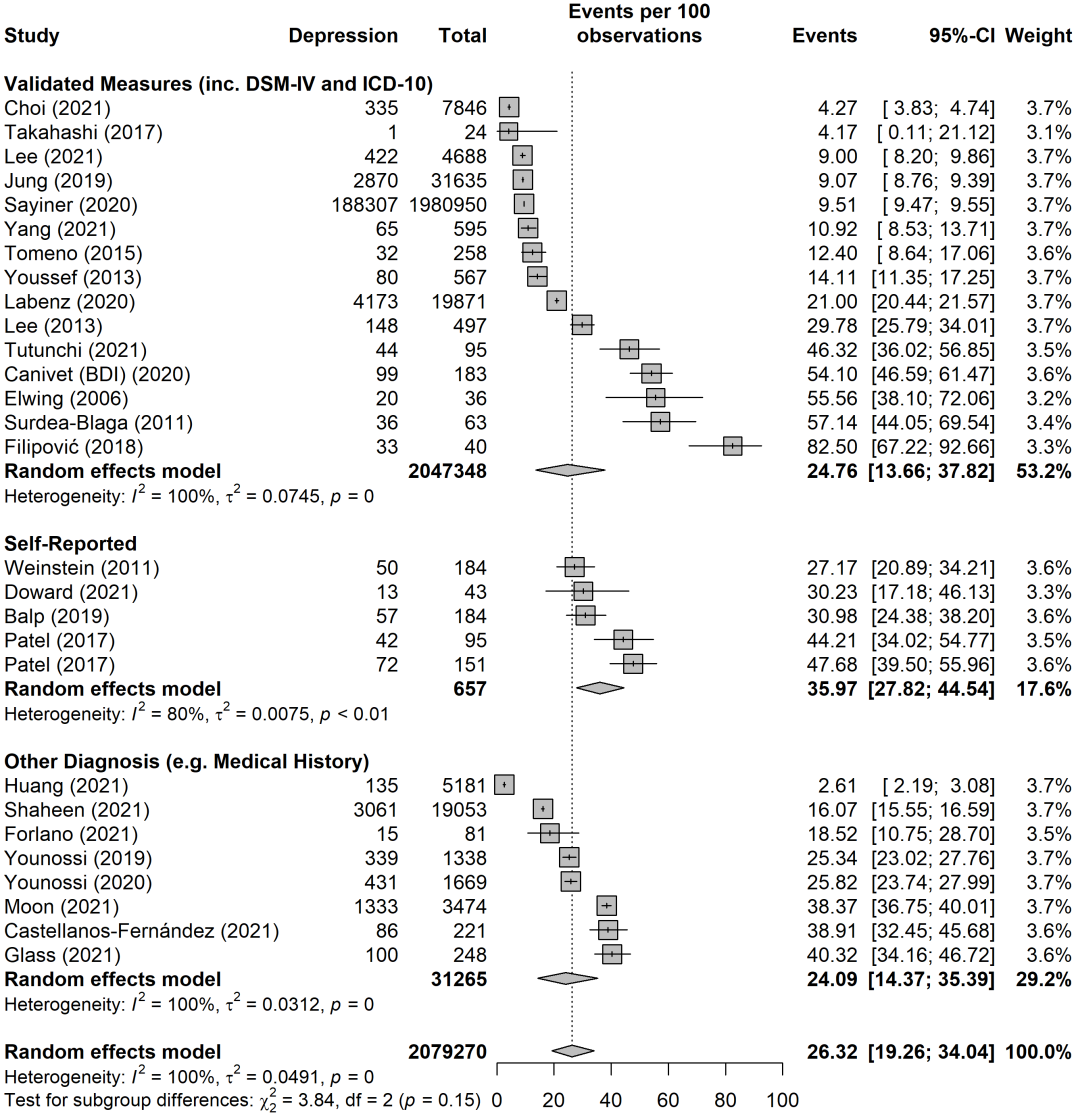
Pooled prevalence of depression, split into subgroups by method of diagnosis, i.e. validated measures (including DSM-IV and ICD-10), self-report, and other diagnosis (e.g. medical history).

The pooled prevalence of depression for all studies yielded an estimate of 26.3% (95% CI: 19.2 to 34%) (**Figure 3**). The *I* ^2^ statistic was 100%, indicating a considerable degree of heterogeneity among the studies. The funnel plot for examination of publication bias is shown in **Supplementary Figure 2**. We found evidence of publication bias as indicated by the asymmetrical funnel plot of studies’ precision against prevalence estimates (in logarithmic scale). However, the results of leave-one-study-out sensitivity analyses showed that no study had undue influence on the pooled depression prevalence as presented in **Supplementary Figure 3A**. The Baujat plot highlighted the study by Sayiner et al. (63) as a significant contributor to the overall heterogeneity and influence on the meta-analysis results (**Supplementary Figure 3B**). The large sample size of this study (63) in relation to the total combined sample size of all studies contributes significantly to the heterogeneity (I² = 100%) of the meta-analysis. Another study by Fillipovic et al. (49) appears to have a minimal influence on the overall meta-analysis result when compared to its contribution to heterogeneity. This suggests that while the study adds to the variability within the meta-analysis, its effect size or weight does not substantially alter the combined effect estimate of depression prevalence.

As presented in **Figure 3**, the pooled estimate tended to be higher among studies that used self-reported tools (36.0%, 95% CI: 27.8 to 44.5%), followed by studies that used validated measures (24.8%, 95% CI: 13.7 to 37.8%), and studies that used other diagnosis such as medical history (24%, 95% CI: 14.3 to 35.3%).

**Figure 4:**
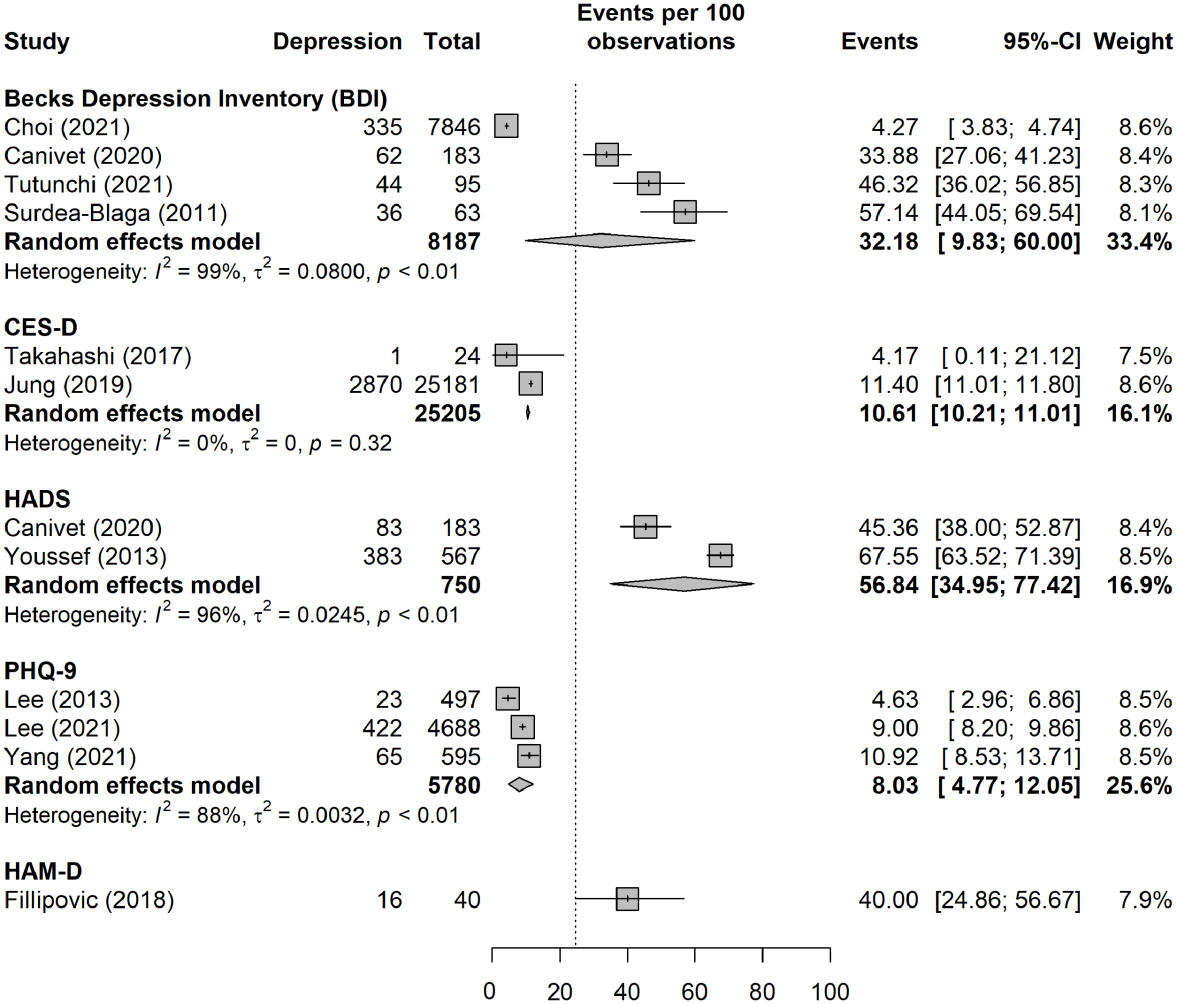
Pooled prevalence of depression, by validated tools/measures. In the sub-group analysis, only data for moderate to severe depression were included for the purpose of consistency across studies.

**Figure 4** presents the results of the meta-analysis stratified by validated tools/measures. The pooled prevalence estimate was highest among studies that used the Hospital Anxiety and Depression Scale (HADS), followed by the Beck Depression Inventory (BDI), the Centre for Epidemiological Studies-Depression (CES-D) scale, and the Patient Health Questionnaire-9 (PHQ-9).

### Anxiety

Of the studies reporting depression, ten additionally measured anxiety, resulting in a total of 12 studies measuring anxiety (43, 45–48, 55, 56, 59, 60, 71–73), with the corresponding total number of participants amounting to 35,034. Validated instruments were used to measure anxiety in four studies (**Table 1**), utilizing the DSM-IV (48), ICD-10 (56), the Hamilton Anxiety Rating Scale (59) and the Hospital Anxiety and Depression Scale (73). Self-report and other diagnosis (e.g., medical history) were used in three (43, 47, 55) and four studies (45, 60, 71, 72), respectively. A further study (46), utilized a validated instrument to measure both state and trait anxiety. To separate the two domains, this study was not incorporated into the primary analysis for pooled prevalence of anxiety and was included in the additional sub-group analyses only.

Six of these studies originated from the USA (47, 48, 60, 71–73), with a total of 7,127 participants. However, the largest number of participants (n = 19,871) was from a study originating from Germany (56).

**Figure 5:**
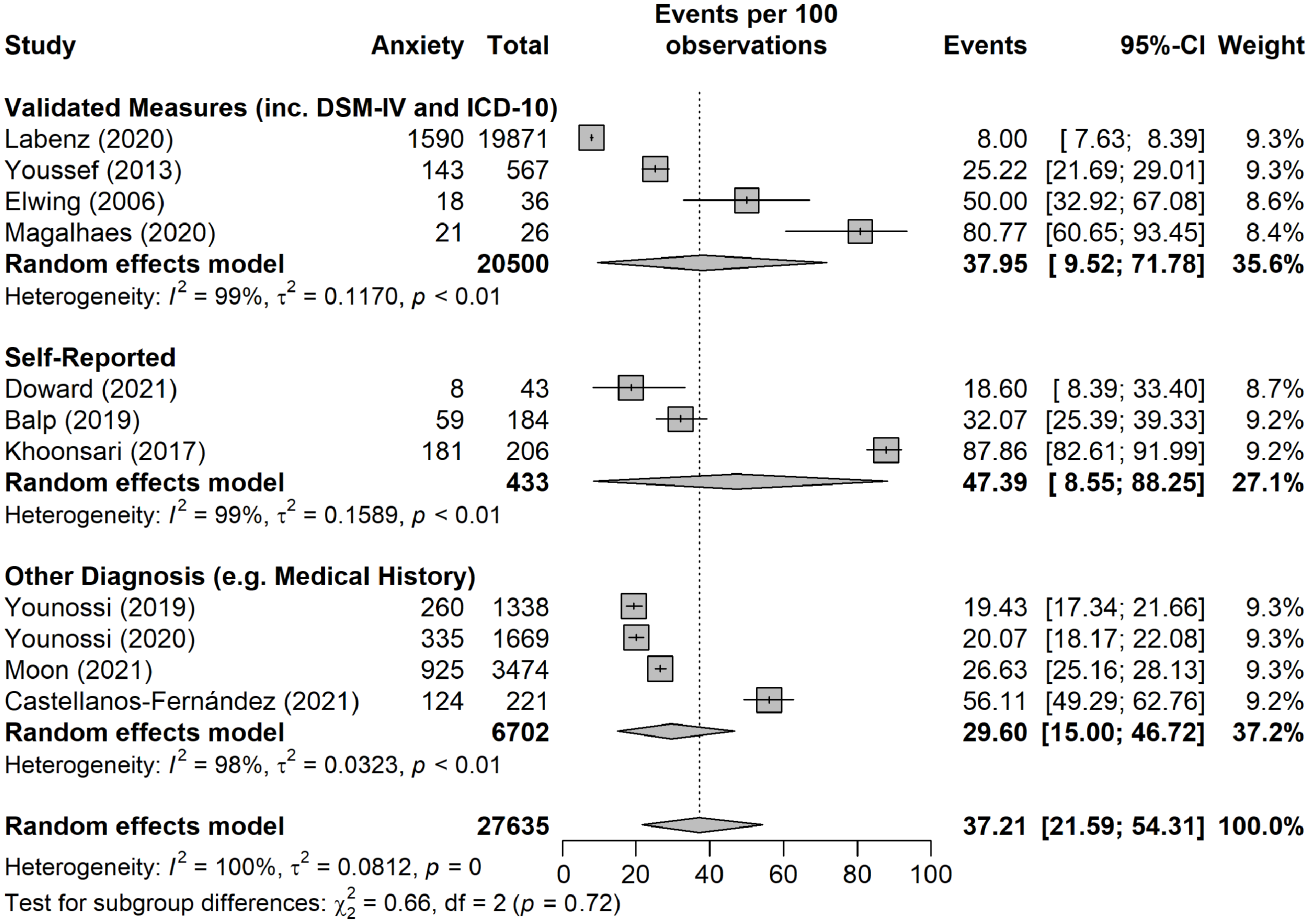
Pooled prevalence of anxiety broken down into subgroups by method of diagnosis, namely validated tool/measure (including DSM-IV and ICD-10), self-report, and other diagnosis (e.g. medical history).

The pooled prevalence of anxiety yielded an estimate of 37.2% (95% CI: 21.6 to 54.3%) (**Figure 5**). As with depression, the *I* ^2^ statistic was 100%, indicating considerable heterogeneity between the studies. The funnel plot for the examination of publication bias is presented in **Supplementary Figure 4**. We found evidence of publication bias as indicated by the asymmetrical funnel plot of studies’ precision against prevalence estimates (in logarithmic scale). However, the results of the leave-one-study-out sensitivity analyses showed that no study had undue influence on the pooled anxiety prevalence (**Supplementary Figure 5**).

As presented in **Figure 5**, the pooled estimate tended to be higher among studies that used self-reported tools (47.4%, 95% CI: 8.5 to 88.2%), followed by studies that used validated measures (38.0%, 95% CI: 9.5 to 71.8%), and studies that used another method for diagnosis such as medical history (29.6%, 95% CI: 15.0 to 46.7).

### Stress

One of the included studies also measured stress in addition to anxiety (59). In total, only two of the included studies investigated stress in association with NAFLD (54, 59), involving a total of 47,564 participants. However, one of these studies (54), conducted in South Korea, included 47,538 participants. Both studies utilized validated instruments to measure stress. One study used the Perceived Stress Inventory to measure stress (54), whilst the other utilized the Lipp’s Stress Symptoms Inventory (59) (**Table 1**).

**Figure 6:**
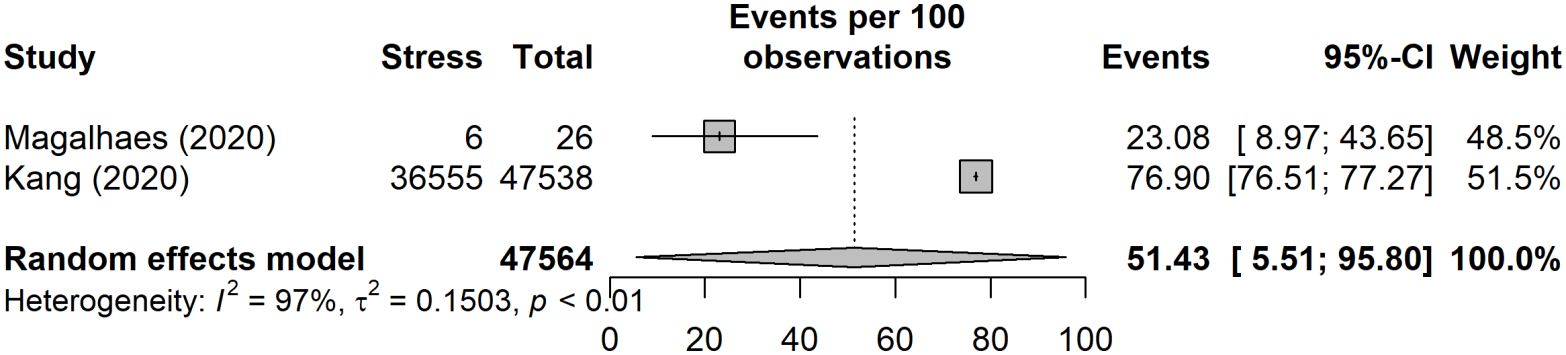
Pooled prevalence of stress.

The pooled prevalence of stress (**Figure 6**) yielded an estimate of 51.4% (95% CI: 5.5 to 95.8%). The *I* ^2^ statistic was 97%, indicating a considerable degree of heterogeneity between the studies.

## Discussion

The present systematic review and meta-analysis presents novel data on the prevalence of depression, anxiety, and/or stress in adults living with NAFLD, whilst comprehensively summarizing the relevant literature. When we meta-analyzed data from 28 studies, a high prevalence of depression was revealed among this patient population (26.3%; 95% CI: 19.2 to 34%). A higher pooled prevalence estimate of 37.2% (95% CI: 21.6 to 54.3%) was noted for anxiety in patients with NAFLD, whilst stress appears to affect one in two patients with NAFLD (51.4%; 95% CI: 5.5 to 95.8%). To our knowledge, this is the largest meta-analysis of available data on the prevalence rates of depression, and/or anxiety, and/or stress among adults with NAFLD, documenting even higher mental health comorbidity in this patient population than previously reported (33). As discussed in the following sections, this apparently high overlap between NAFLD and these common mental health problems constitutes a significant health issue which merits further attention both in the context of the clinical care of these patients and for targeted research in this field.

### Depression

Depression is a highly prevalent disorder worldwide, constituting a leading cause of years lived with disability and affecting over a quarter of a billion people (74). The findings of this systematic review suggest that depression is present in approximately one out of four (~26.3%) patients with NAFLD. This prevalence of depression is even higher than the one reported in a previous meta-analysis (33), which included 10 studies with an 18.21% pooled prevalence of depression in patients with NAFLD. This may, at least in part, be reflective of the larger number of studies and the larger sample size included in our systematic review.

Notably, in the larger study included in the present systematic review, involving 1,980,950 Medicare beneficiaries, depression was reported to be one of the most common extra-hepatic diseases identified in people with NAFLD (63). Depression was further reported as a contributing factor to impaired health-related quality of life (45, 52), whilst there are data supporting an independent association of depression with high-risk behaviors, such as sedentariness and unhealthy diet among individuals with NAFLD (51). Another included study reported similarities between both men and women regarding a significant association between NAFLD and the incidence of depression, independently of other confounders such as diabetes, CVD, asthma, sex and age (56). In addition, a further retrospective cross-sectional study conducted by Choi et al. (46), involving 7,846 participants, identified an independent association of NAFLD with the risk for depression after controlling for other factors including diabetes and age.

Regarding more severe forms of NAFLD, studies investigating rates of depression in patients with NASH identified a higher frequency of depression among this patient group (43, 47, 48). Additional evidence from included studies suggests an association of depression with NAFLD progression/severity, with the study by Tomeno et al. (67) showing that major depressive disorder was associated with more severe histological hepatic steatosis and worse treatment outcomes in patients with NAFLD. Furthermore, both major depressive disorder and general anxiety disorder have been identified as being significantly increased in patients with NASH, and associated with increased advanced liver histological abnormalities (48). An association of increased symptoms of depression with a greater likelihood of severe hepatocyte ballooning has also been reported by Youssef et al. (73).

Contrary to the above, one study reported that depression was not found to be independently associated with NAFLD at a population level after controlling for other confounding factors, such as diabetes and obesity (58). Likewise, in a study conducted in Romania, authors reported that they were unable to detect a relationship between NAFLD and depression and anxiety, highlighting that symptoms of depression and anxiety are common in this particular region (65).

### Anxiety

A total of 12 studies included in the present systematic review measured anxiety, resulting in a pooled prevalence rate of ~37.2%. Thus, as with depression, our findings indicate that anxiety appears to be a very common mental health problem among patients with NAFLD, which has a potential impact on the overall health-related quality of life (43, 45, 47, 55). Notably, one study indicated that general anxiety disorder is significantly increased in patients with NASH and is associated with advanced liver histological abnormalities (48).

Furthermore, the study by Choi et al. (46) explored the presence of both state anxiety and trait anxiety among a NAFLD population, demonstrating that, although NAFLD in itself was not significantly associated with anxiety, associations with state and trait anxiety did emerge depending on the stage of steatosis. These associations remained consistent after adjusting for factors such as age, body mass index (BMI), diabetes, and smoking, but were evident only in females. However, a study by Magalhaes et al. (59) did not identify significant associations between NAFLD and anxiety, although all participants with NAFLD had some level of anxiety.

### Stress

Growing evidence suggests an association between chronic psychosocial stress and an increase in the prevalence of various cardio-metabolic diseases, such as obesity, T2DM, CVD and hypertension (75, 76). Despite this emerging importance of chronic stress as a potential factor associated with metabolic syndrome and NAFLD, there is a paucity of studies which have explored such a relationship. Indeed, the present systematic review identified only two eligible studies which investigated chronic stress in relation to NAFLD, gleaning a pooled prevalence of ~51.4%. Interestingly, of these two studies, the large cross-sectional study conducted in South Korea (54) identified a positive independent association between increased prevalence of NAFLD and perceived stress, suggesting a probable relationship between the two. Contrarily, the small study by Magalhaes et al. (59), which sought to identify an association between NAFLD and occupational stress among 26 healthcare professionals employed at a community hospital in Brazil, failed to confirm a significant relationship between stress and the presence of NAFLD, although the authors suggest that such an association should continue to be explored. Accordingly, caution should be adopted when interpreting these findings since data are drawn from only two studies. However, this (both the existing data and the absence of more such data) should clearly prompt further research into the potential links between NAFLD and chronic stress.

### Comparison with other population groups and general population data

Certain studies included within this review investigated the prevalence of mental health problems in patients with NAFLD compared with other population groups (48, 49, 68–70). For example, the study by Elwing et al. (48) identified a higher rate of depression and anxiety in patients with NASH compared to a matched control group without liver disease. Furthermore, Fillipovic et al. (49) demonstrated greater risk of cognitive impairment and depression in patients with NAFLD compared to those without, whilst Weinstein et al. (69) reported a higher prevalence of depression in individuals with NAFLD in comparison with patients with another liver disease, namely those with a hepatitis B virus infection.

In terms of comparisons with general population data, data suggest a lifetime prevalence estimate for depression of 14.6% and an average 12-month prevalence estimate of 5.5% for adults in high-income countries (77). It is further estimated that generalized anxiety disorder has a lifetime prevalence of between 1-7% in Europe and around 7.8% in the USA, although it is suggested that generalized anxiety disorder is often underdiagnosed (78). Therefore, based on even the lower corresponding estimates from the present systematic review, it appears that the prevalence of depression and anxiety among patients with NAFLD is likely to be considerably higher when compared to the general population.

Depression is a key health issue of concern globally, which has significantly worsened after the COVID-19 pandemic, with the WHO reporting that the prevalence of depression and anxiety increased by 25% within the first year of this pandemic (79). Furthermore, depression is reported to be a common co-existing problem among patients with chronic disorders (80). For example, a large prospective cohort study conducted in Spain, identified that around 20% of patients with diabetes suffered from depression, and that this was associated with a number of diabetes related outcomes and complications (81). A further systematic review revealed a 28% prevalence of anxiety in patients with diabetes, with those with pre-existing anxiety at higher risk of developing diabetes (19%) (82). Likewise, stress is reported as a trigger for the onset of both type 1 and type 2 diabetes, with the combination of chronic stress and obesity leading to metabolic failure and increasing diabetes risk in such individuals (83). Depression, anxiety and chronic psychological stress are also reported as being common in people with CVD, with a recent systematic review revealing a prevalence of depression at 31.3%, and anxiety and stress at 32.9% and 57.7%, respectively, among this population (84). Moreover, a systematic review and meta-analysis by Mejarah et al. (83), revealed a high prevalence of depression among cancer patients, with the highest prevalence being identified among those with colorectal cancer (32%) (83), whilst a 13.8% prevalence of anxiety among patients with cancer has also been reported (85).

Thus, our present findings suggest that the prevalence rates of these common mental health problems in patients with NALFD may be similar to those documented for other chronic disorders; however, this seems to have received less attention and awareness among the NAFLD population in comparison to other patient groups.

### Diagnosis of NAFLD/measurement of mental health

Among the studies included in this review, a range of methods were used to diagnose NAFLD. In general, liver biopsy continues to be considered the gold standard for the diagnosis of NAFLD and NASH, as it allows the histologic assessment of hepatic steatosis, inflammation, and fibrosis. However, liver biopsy is an invasive strategy which is costly, not always feasible, and carries a risk of complications (e.g., bleeding). As such, many patients are currently diagnosed via non-invasive methods (e.g., ultrasound and other imaging methods), with liver biopsy more commonly reserved for use where there is diagnostic uncertainty (86, 87). This also explains the range of NAFLD diagnostic methods utilized in the studies included in this systematic review (**Table 1**).

Regarding assessment of the mental health problems of interest, a number of the included studies involved the use of well-established validated tools/methods, whilst others utilized self-report or other means, such as medical records. Of interest, for studies where depression and anxiety were self-reported, a higher prevalence of these conditions was evident - a finding that was also noted in a previous systematic review (33). This may be due to problems regarding patient recall of physician diagnosis, but might also reflect the possibility that generic validated tools may not capture depression and anxiety among this specific NAFLD patient group. To our knowledge, there are no mental health measures validated specifically for NAFLD patients. Likewise, as far as we are aware, the tools that are currently in widespread use for measuring common mental health problems have not been specifically validated for use among this patient group.

It is important to note that in some of the studies included in this review, mental health was not the primary outcome. For example, two of our included studies had a focus on binge eating disorder (BED), with the primary aim being to assess if BED related to obesity was associated with the severity of NAFLD in one study (44), and to assess risk factors for the presence of BED among patients with NAFLD together with the impact of BED on body mass composition in another study (50). Additionally, Patel et al. (61) sought to describe the number and type of chronic conditions and medications taken by patients with diabetes and NAFLD and to identify characteristics that may impact on liver disease severity, whilst another study by Patel et al. (62) aimed to examine the association between lifetime alcohol consumption and significant liver disease in patients with diabetes and NAFLD (62). Therefore, the assessment of mental health might be seen as a secondary objective of these studies, and, thus, care should be taken when interpreting these findings, since the relevant mental health issues identified might be due to other causes beyond NAFLD itself. However, it should be noted that when these studies were omitted during the performed leave-one-out sensitivity analysis, their omission had no significant effect on the overall pooled prevalence of depression.

### Potential underlying mechanisms

The present systematic review specifically looked at the prevalence of one or more common mental health issues (i.e., depression, anxiety and stress) in adults with NAFLD, thus the included studies offered evidence predominantly on this research question. However, growing broader data suggest that a bi-directional pathophysiologic association between NAFLD and depression might be in existence (31), whilst it is also plausible that a feed-forward vicious cycle exists between these common mental health conditions and NAFLD, whereby such mental health morbidity may promote NAFLD, and *vice versa* (12). Thus, it is important to consider the potential underlying mechanisms that may link NAFLD with these common mental health problems. Indeed, some of the studies included in this review also refer to such potential underlying mechanisms, including insulin resistance, inflammation, and the activity of the hypothalamic-pituitary-adrenal axis (HPA) axis (46, 54, 56, 73). For example, when exploring the association between depression and NAFLD, one of the studies included within this systematic review suggests that insulin resistance appears to play an important role in modulating the link between depression and NAFLD risk (57). Moreover, the potential involvement of the serotonin pathway, and the gut microbiome have also been discussed in the context of underlying mechanisms linking NALFD and these mental health problems (12, 46). Finally, brain insulin resistance, neuro-inflammation and cerebrovascular changes are also considered as part of the NAFLD-related pathophysiology which may affect the central nervous system in these patients and could contribute to the development of depression and anxiety (88). Of note, a Mendelian randomization study by Lin et al. showed that NAFLD causally affects the brain cortical structure, revealing an association between NAFLD (NAFLD activity score and fibrosis stages) and cortical structures (reduced global surface area and changes in the cortical structures of several brain gyri as assessed by MRI) which may contribute to disease/dysfunction of the central nervous system (89). These findings further support the notion of a liver-brain axis and suggest that MRI scans could be introduced in the routine care offered to patients with NAFLD in order to promptly diagnose potential neuropsychiatric comorbidity (89).

It is important to highlight that there could be many other factors that may contribute to the mental health and well-being of patients with NAFLD, including symptoms of fatigue which may impact on quality of life and the high risk of significant complications, as well as the lack of awareness of the condition and perceived stigmatization (28). Furthermore, it is reported that NAFLD patients with depression are at a greater risk of adverse outcomes, such as stroke, CVD and cancer-related mortality compared to those without depression (90). Similarly, anxiety has been shown to be associated with a number of health issues including CVD, hypertension and gastrointestinal issues (91), and increased levels of anxiety among NAFLD patients might also lead to further physical complications. Anxiety may also impair quality of life both in terms of physical and mental health and in association with everyday functioning (92), and this is highly likely to be the case with NAFLD patients.

Overall, NAFLD is a complex condition and may further be associated with various socioeconomic factors and unmet needs, which could in turn lead to mood imbalances and feelings of social isolation and loneliness, representing a further substantial risk to overall health and quality of life (28). Interestingly, chronic loneliness is reported as being associated with both mental health problems and metabolic disorders, potentially acting as a chronic stressor leading to HPA axis overactivity which may contribute to the development of both mental health and metabolic problems that, in turn, may also lead to feelings of social isolation (93).

### Limitations

This systematic review and meta-analysis has certain limitations. Firstly, because a number of the included studies were cross-sectional in design, it is not possible to determine causality. In addition, our analysis included some studies wherein mental health issues were not representative of the intended primary study outcomes. Also, high heterogeneity was documented throughout the analysis, which is potentially due to the cross-sectional nature of many of the included studies, different methods used for diagnosing NAFLD and measuring mental health, and differences across country of study origin, and sample size. High levels of heterogeneity have also been identified in previous reviews of this nature (31, 33). It is possible that high heterogeneity is a common feature in meta-analyses of observational studies, due to high risk of bias and because not all included studies may be answering the same research question (94). In terms of risk of bias judgement for the studies included in our review, risk of bias was judged highest for the selection bias domain, since 13 of the included studies involved patients recruited from a single center. These centers were predominantly either liver clinics or centers specializing in gastroenterology or hepatology, implying that the corresponding findings may not be representative of the general population of patients with NAFLD. In addition, in the present systematic review we included only papers which were published in the English language, whilst we did not include unpublished studies. Hence, it is likely that there may be additional relevant studies which are currently unpublished or have been published in languages other than English. Furthermore, it was not possible to explore potential ethnicity related differences in the context of this systematic review since ethnic specific data were not consistently reported by the included studies. It would be of interest if future research could further investigate differences in the prevalence, disease management, and associations of NAFLD and mental health problems across different ethnic groups. Finally, it was not possible to further analyse potential differences depending on the exact stage of NAFLD and whether steatosis/steatohepatitis and/or comorbid conditions are present or not, since the included studies did not consistently report such detailed data as well.

### Concluding remarks

Given that the prevalence rates of both NAFLD and mental health problems are expected to continue to increase globally, a further growth in the patient group presenting with such comorbid chronic problems should be expected in the following years. Thus, it is important for the clinical practice to ascertain the exact degree of mental health comorbidity among the NAFLD patient population in order to prioritize and/or tailor relevant treatment interventions. The present systematic review and meta-analysis presents such up-to-date data on the apparently high prevalence of depression, anxiety, and stress among adults with NAFLD, and comprehensively summarizes the existing relevant literature. Our findings show markedly high pooled prevalence rates of these mental health disorders in adults with NAFLD, indicating a plausible underlying pathophysiological link, however, the present work does not draw conclusions on such an association. Thus, additional research is required to elucidate the potential pathophysiological links between these common mental health disorders and NAFLD, and to further identify the exact risk of developing stress, anxiety and depression disorders in this patient population. Indeed, our present work further highlights such gaps/weaknesses which remain within the relevant literature, including the need to understand potential bi-directional links between NAFLD and mental health problems. Therefore, whilst clinical practice should acknowledge the apparently high prevalence rates of depression, anxiety, and stress among adults with NAFLD and accordingly offer tailored care to these patients, research efforts should also be directed on elucidating potential underlying mechanisms shared between these common chronic health problems which could result in developing novel treatment options for such patients.

## Data availability statement

Information for existing publicly accessible datasets is contained within the article.

## Author contributions

SS: Conceptualization, Formal analysis, Visualization, Writing – original draft, Writing – review & editing. CL: Conceptualization, Visualization, Writing – review & editing. CK: Formal analysis, Visualization, Writing – review & editing. LL: Visualization, Writing – review & editing. OU: Formal analysis, Visualization, Writing – review & editing. AD: Formal analysis, Visualization, Writing – review & editing. LA: Supervision, Visualization, Writing – review & editing. SC: Visualization, Writing – review & editing. HR: Conceptualization, Supervision, Visualization, Writing – review & editing. IK: Conceptualization, Supervision, Visualization, Writing – review & editing.
