# Supplementary material for: Non-alcoholic fatty liver disease and coexisting depression, anxiety and/or stress in adults: a systematic review and meta-analysis
Source: Front Endocrinol (Lausanne). 2024 Apr 16;15:1357664. doi: 10.3389/fendo.2024.1357664 (PMC11058984; doi:10.3389/fendo.2024.1357664)

**Supplementary Figure 1.** Traffic Light Plot - Judgement regarding each domain of the risk of bias assessment for non-randomized studies (RoBaNS) for all included studies.


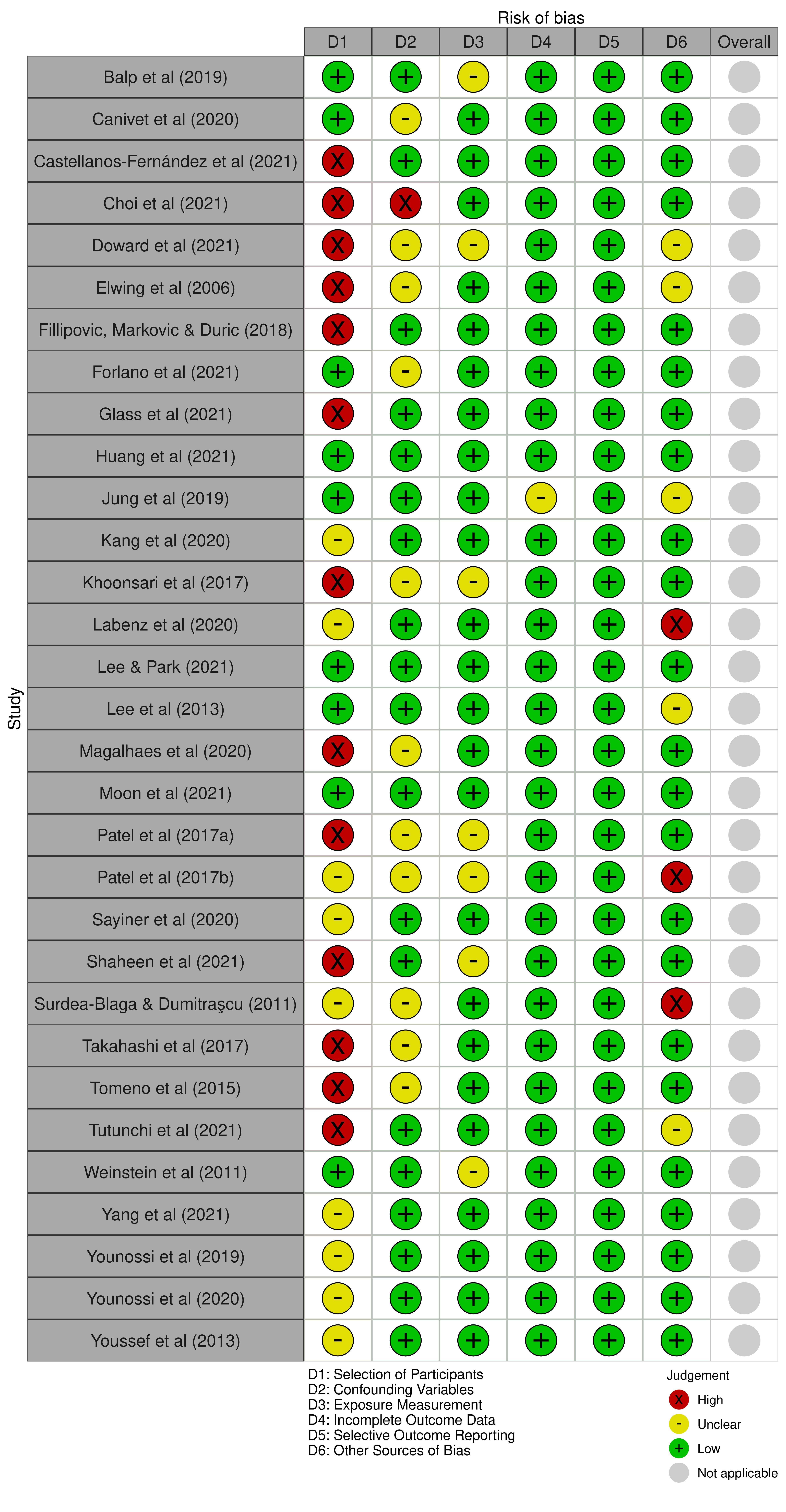


**Supplementary Figure 2:** Funnel plot for depression prevalence estimates.

**
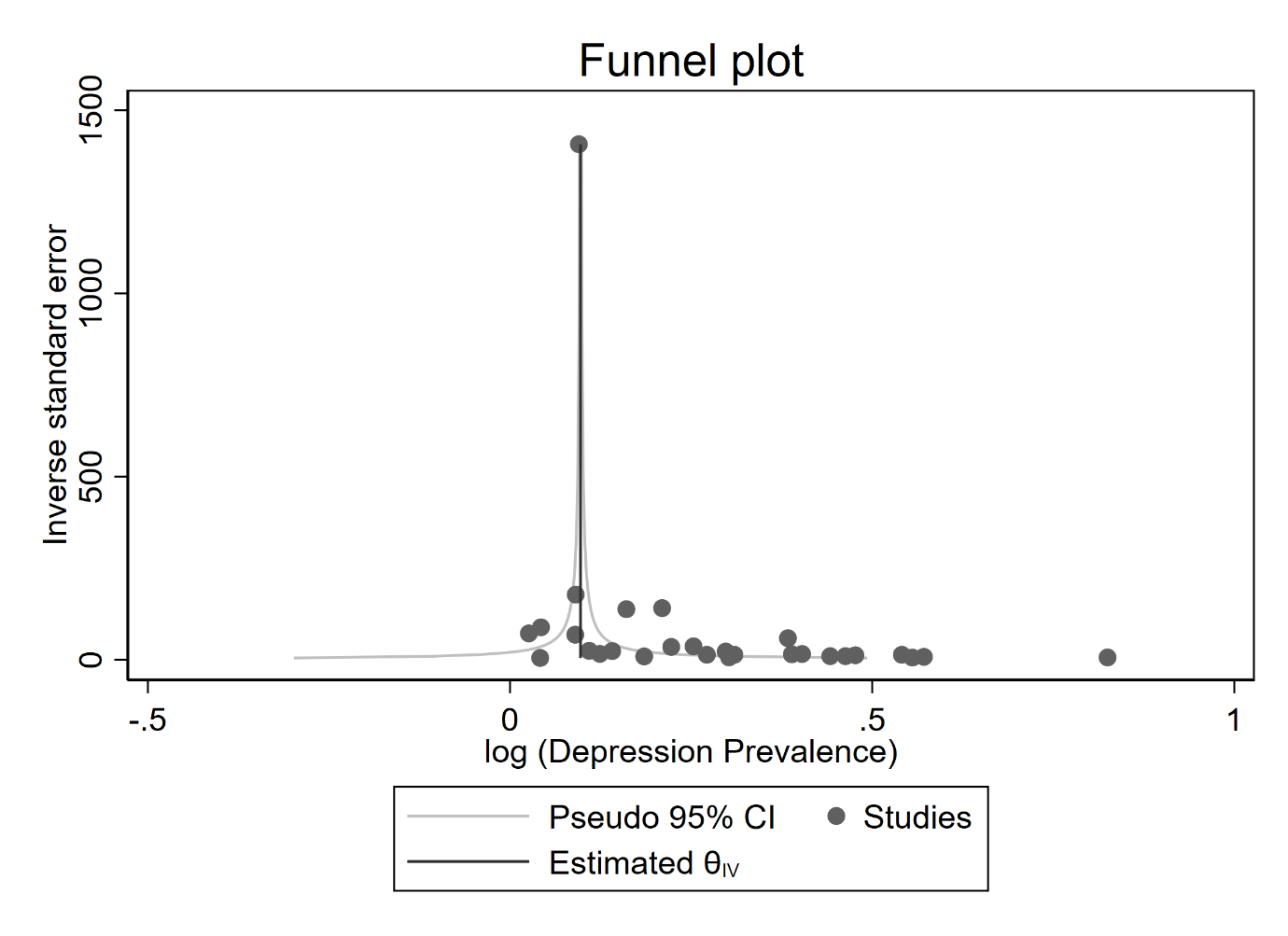
**

**Supplementary Figure 3. (A)** Leave-one-out sensitivity analyses for depression prevalence estimates**; (B)** Baujat plot analysis for evaluating study influence and heterogeneity in meta-analysis results.

**(A)**


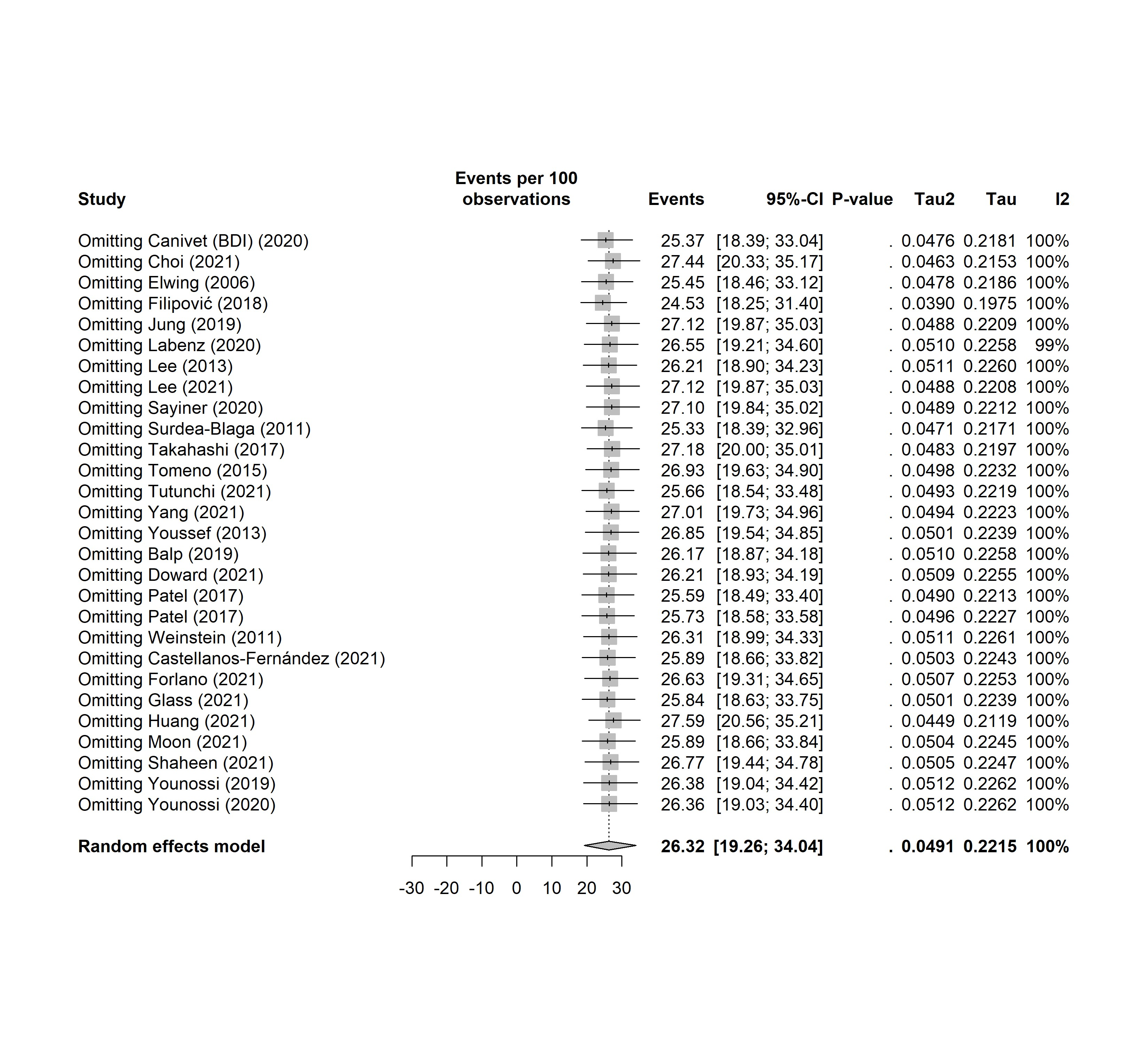


**(B)**
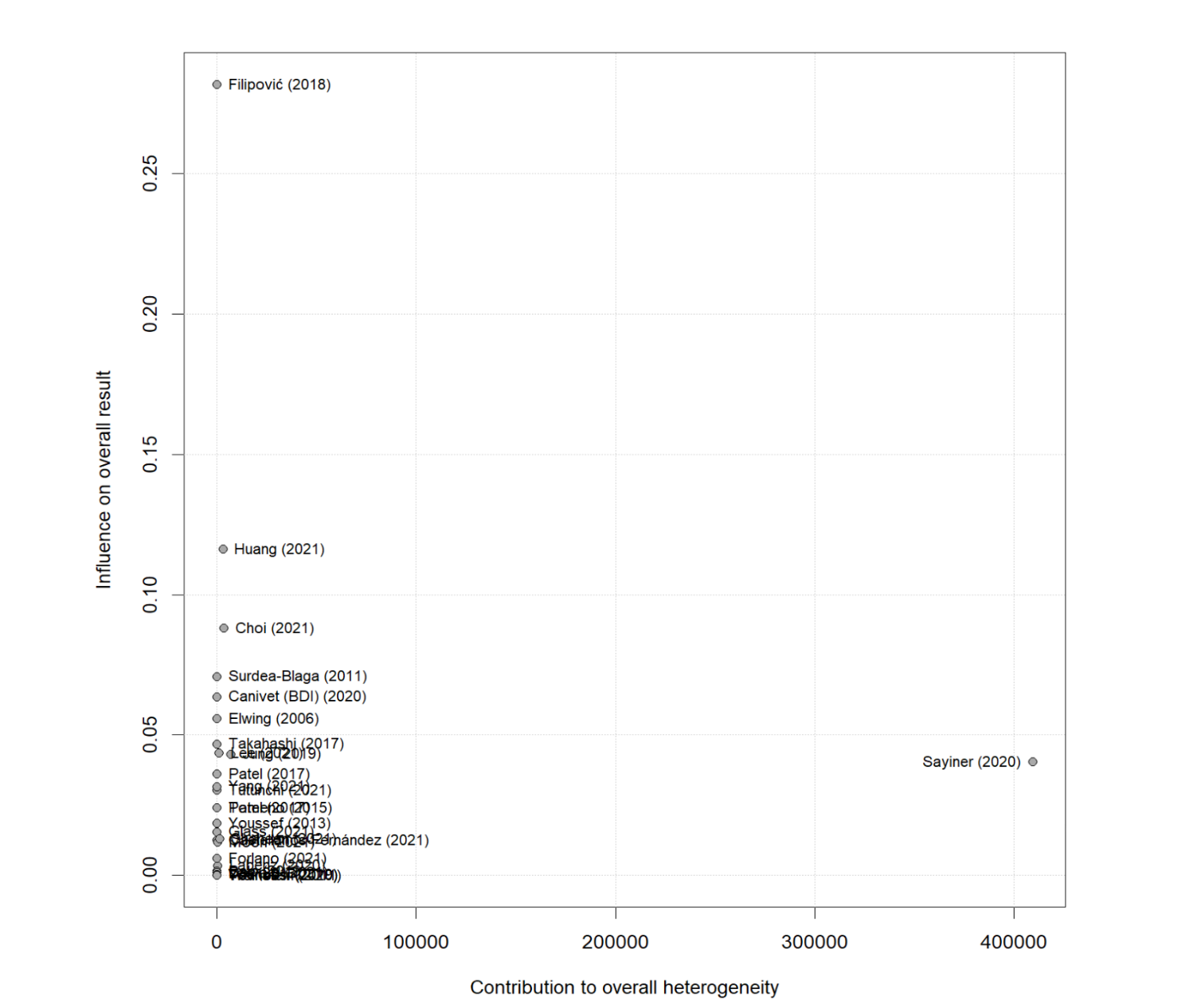


**Supplementary Figure 4:** Funnel plot for anxiety prevalence estimates.

**
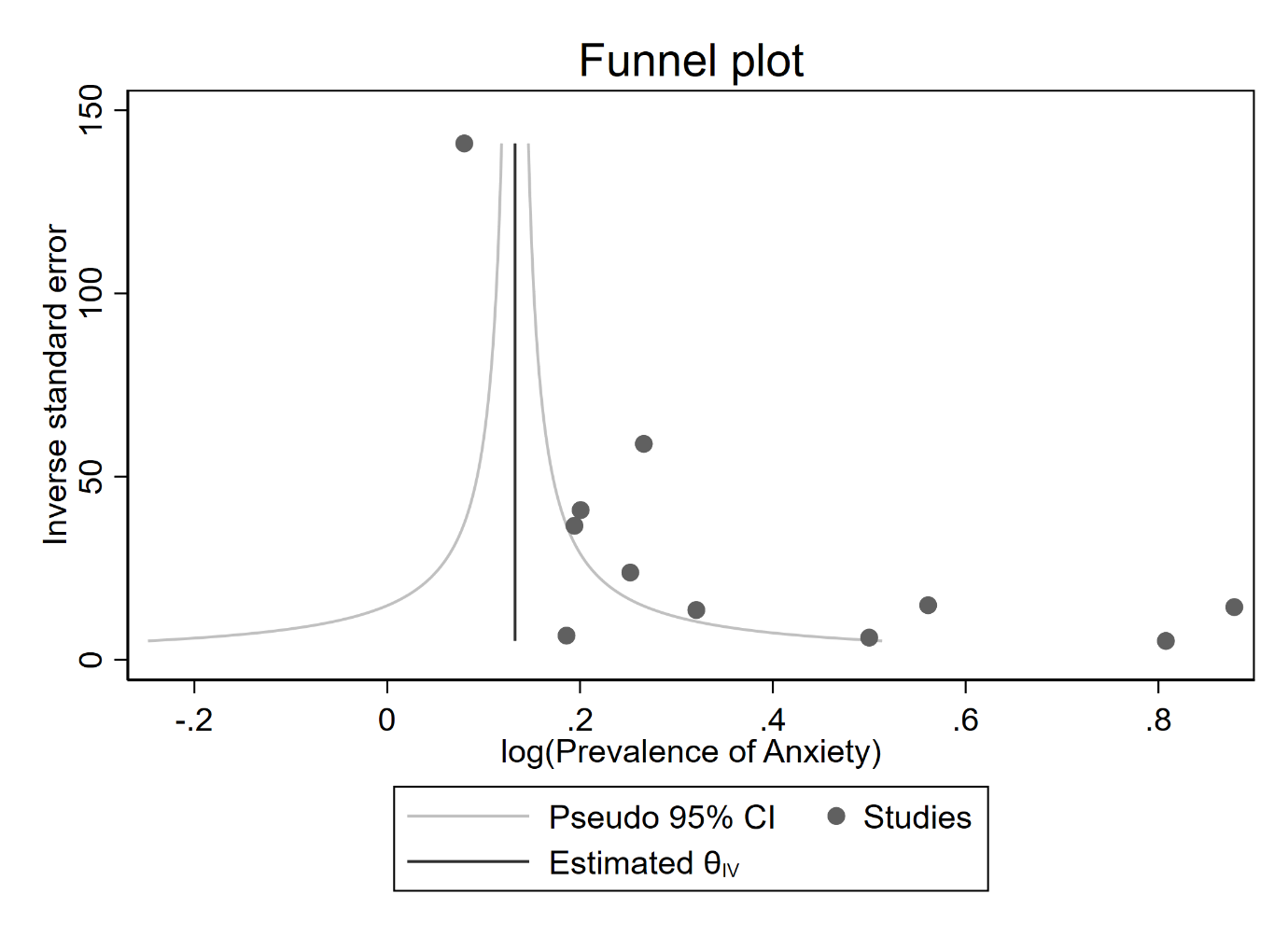
**

**Supplementary Figure 5:** Leave-one-out sensitivity analyses for anxiety prevalence estimates
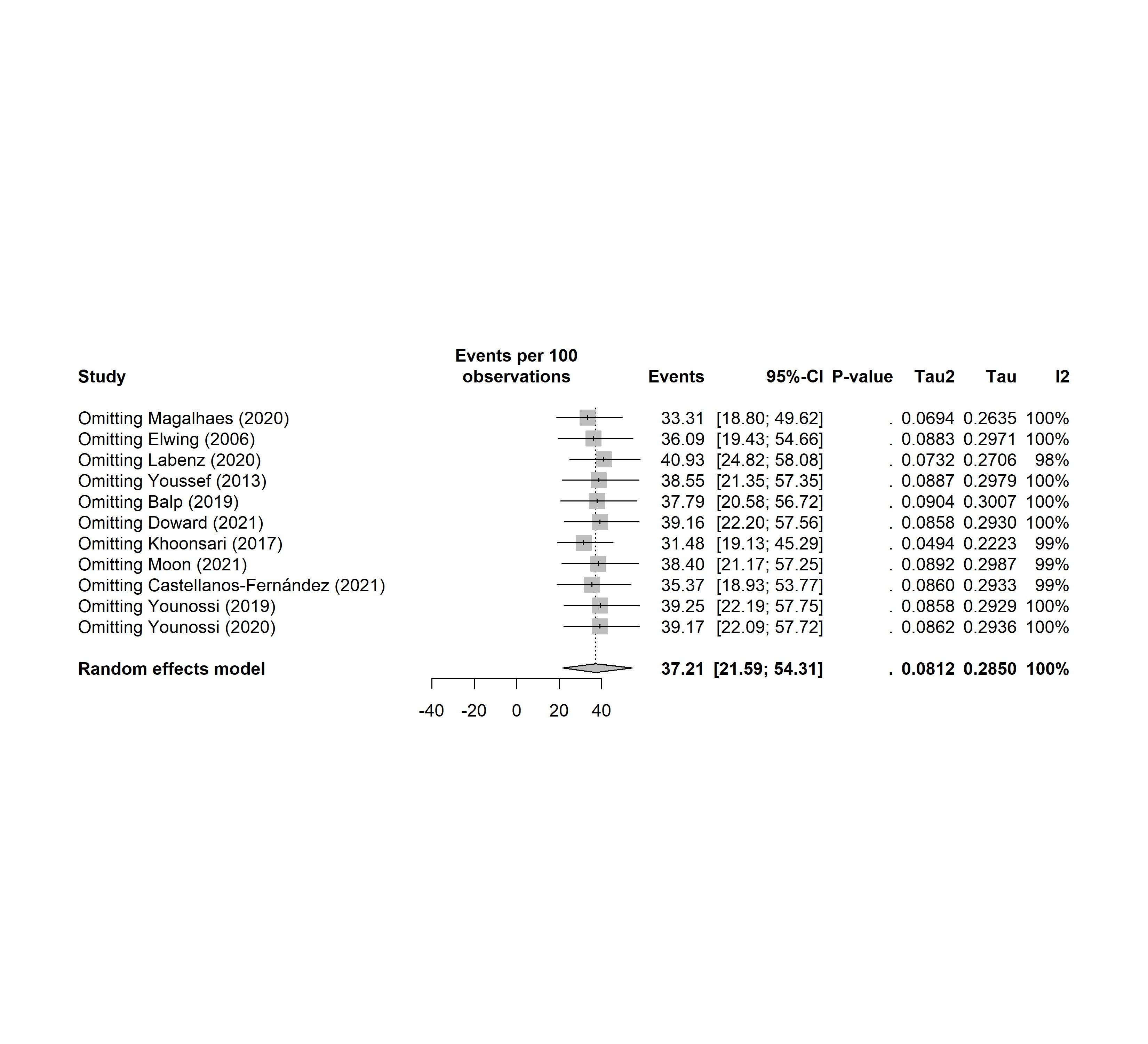

Supplement: Supplementary file 1 [file DataSheet_1.docx]
