# Supplementary material for: Non-alcoholic fatty liver disease and coexisting depression, anxiety and/or stress in adults: a systematic review and meta-analysis
Source: Front Endocrinol (Lausanne). 2024 Apr 16;15:1357664. doi: 10.3389/fendo.2024.1357664 (PMC11058984; doi:10.3389/fendo.2024.1357664)
Supplement: Supplementary file 2 [file Table_1.docx]

**Supplementary Table S1 – Search Strategy**

**PubMed**

((metabolic associated fatty liver disease[Title/Abstract] OR MAFLD OR metabolic dysfunction associated fatty liver disease[Title/Abstract] OR NAFLD[MeSH Terms] OR NAFLD OR non-alcoholic fatty liver disease[Title/Abstract] OR non-alcoholic steatohepatitis[Title/Abstract] OR NASH)) AND ((mental health[MeSH Terms] OR mental health[Title/Abstract] OR "mental health" OR "mental well-being" OR "mental wellbeing" OR depression[MeSH Terms] OR depression[Title/Abstract] OR major depressive disorder[MeSH Terms] or major depressive disorder[Title/Abstract] OR major depression[Title/Abstract] OR MDD OR anxiety[MeSH Terms] OR anxiety[Title/Abstract] OR generalized anxiety disorder[MeSH Terms] OR generalized anxiety disorder[Title/Abstract] OR generalised anxiety disorder[Title/Abstract] OR stress, psychologic[MeSH Terms] OR disorder, mood[MeSH Terms] OR distress[Title/Abstract]))

**EBSCOhost**
( (TI “metabolic associated fatty liver disease”) OR (AB “metabolic associated fatty liver disease”) OR MAFLD OR (MH "Nonalcoholic Fatty Liver Disease") OR NAFLD OR (TI "Non-alcoholic Fatty Liver Disease") OR (AB "Non-alcoholic Fatty Liver Disease") OR (TI “Non-alcoholic steatohepatitis”) OR (AB “Non-alcoholic steatohepatitis”) OR NASH ) AND ( (MH “Mental Health”) OR (TI “Mental Health”) OR (AB “Mental Health”) OR “Mental Health” OR “mental well-being" OR “mental wellbeing” OR (MH “Depression”) OR (TI “Depression”) OR (AB ”Depression”) OR (TI ”Major depressive disorder”) OR (AB “Major depressive disorder”) OR (TI “Major depression”) OR (AB ”Major depression”) OR MDD OR (MH “Anxiety”) OR (MH “Generalized Anxiety Disorder”) OR (TI “Anxiety”) OR (AB “Anxiety”) OR (TI “Generalized Anxiety Disorder”) OR (AB “Generalized Anxiety Disorder”) OR (TI “Generalised Anxiety disorder”) OR (AB “Generalised Anxiety Disorder”) (MH “Stress, Psychological”) OR (TI “Distress”) OR (AB “distress”) )

**ProQuest**

((mesh.Exact("non alcoholic fatty liver disease") OR ti(“NAFLD”) OR ab(“NAFLD”) OR ti(“metabolic associated fatty liver disease”) OR ab(“metabolic associated fatty liver disease”) OR ti(“Non alcoholic steatohepatitis”) OR ab(“Non alcoholic steatohepatitis”) OR ti(NASH)) OR ab(NASH)) AND (mesh.Exact("mental health") OR ti(“mental health”) OR ab(“mental health”) OR “mental health” OR “mental well-being” OR “mental wellbeing” OR SU.exact(“depressive disorder”) OR SU.exact(“depression”) OR ti(“major depression”) OR ab(“major depression”) OR (“major depressive disorder”) OR ab(“major depressive disorder”) OR ti(MDD) OR ab(MDD) OR SU.exact(“generalized anxiety disorder”) OR ti(“generalized anxiety disorder”) OR ab(“generalized anxiety disorder”) OR ti(“generalised anxiety disorder”) OR ab(“generalised anxiety disorder”) OR SU.exact(“psychological stress”) OR ti(“distress”) OR ab(“distress”) OR SU.exact(“mood disorders”))

**Ovid**

nonalcoholic fatty liver/ or ("metabolic associated fatty liver disease" or "metabolic-associated fatty liver disease" or MAFLD or "metabolic dysfunction associated fatty liver disease" or "non-alcoholic steatohepatitis" or "non alcoholic associated steatohepathisis" or NASH).ab,ti.

AND

“depression" or "major depression" or "major depressive disorder" or MDD or anxiety or "generalised anxiety disorder" or "generalized anxiety disorder" or "distress mood disorders").ab,ti.

**Web of Science**

TS=("non-alcoholic fatty liver disease") OR TS=(NAFLD) OR TI=("non-alcoholic fatty liver disease") OR AB=("non-alcoholic fatty liver disease") OR TI=("metabolic associated fatty liver disease") OR AB=("metabolic associated fatty liver disease") OR TI=(metabolic dysfunction associated fatty live disease) OR AB=(metabolic dysfunction associated fatty liver disease) OR TI=(NAFLD) OR AB=(NAFLD) OR TI=("non-alcoholic steatohepatitis") OR AB=("non-alcoholic steatohepatitis") OR TI=(NASH) OR AB=(NASH)

AND

TS=("mental health") OR TI=("mental health") OR AB=("mental health") OR TI=("mental wellbeing") OR AB=("mental wellbeing") OR TI=("mental well-being") OR AB=("mental well-being") OR TS=(depression) OR TI=(depression) OR AB=(depression) OR TS=("major depression") OR TI=("major depression") OR AB=("major depression") OR TS=("major depressive disorder") OR TI=("major depressive disorder") OR AB=("major depressive disorder") OR TI=(MDD) OR AB=(MDD) OR TS=("generalized anxiety disorder") OR TI=("generalized anxiety disorder") OR AB=("generalized anxiety disorder") OR TS=("generalised anxiety disorder") OR TI=("generalised anxiety disorder") OR AB=("generalised anxiety disorder") OR TS=("psychological stress") OR TI=("psychological stress") OR AB=("psychological stress") OR TI=(distress) OR AB=(distress) OR TS=("mood disorder")

Document Types: Articles

**Scopus**

TITLE-ABS-KEY ( "non-alcoholic fatty liver disease" OR "NAFLD" OR "nonalcoholic fatty liver disease" OR "non alcoholic fatty liver disease" OR "NASH" OR "nonalcoholic steatohepatitis" OR "non alcoholic steatohepatitis" OR " non-alcoholic steatohepatitis" OR "metabolic associated fatty liver disease" OR "MAFLD" OR "metabolic associated fatty liver disease" ) AND TITLE-ABS-KEY ( "mental health" OR "mental wellbeing" OR "mental well-being" OR depress* OR "major depressive disorder" OR "major depression" OR "MDD" OR "anxiety" OR "generalized anxiety disorder" OR "generelised anxiety disorder" OR "psychological stress" OR "mood disorder" )
